# *BRCA1* and *BRCA2* Testing through Next Generation Sequencing in a Small Cohort of Italian Breast/Ovarian Cancer Patients: Novel Pathogenic and Unknown Clinical Significance Variants

**DOI:** 10.3390/ijms20143442

**Published:** 2019-07-12

**Authors:** Paola Concolino, Gianfranco Gelli, Roberta Rizza, Alessandra Costella, Giovanni Scambia, Ettore Capoluongo

**Affiliations:** 1 Dipartimento Scienze di Laboratorio e Infettivologiche, Fondazione Policlinico Universitario Agostino Gemelli, IRCCS, 00168 Rome, Italy; 2 Ambulatorio Genetica Medica e Citogenetica Clinica, Poliambulatorio Sant’Anna, ASL Roma 1, Via Garigliano 55, 00198 Rome, Italy; 3 Department Woman and Child Health Sciences, Catholic University of the Sacred Heart, 00168 Rome, Italy; 4 Dipartimento di Medicina Molecolare e Biotecnologie Mediche Università Federico II, CEINGE Biotecnologie Avanzate, 80145 Naples, Italy

**Keywords:** molecular diagnosis, genetic testing, breast cancer, ovarian cancer, NGS, *BRCA1/2* genes

## Abstract

The aim of this report is to describe results of *BRCA1* and *BRCA2* Next Generation Sequencing Analysis (NGS) analysis in 132 selected Italian patients with breast/ovarian cancer. A NGS pipeline with a reliable Copy Number Variation (CNV) prediction algorithm was applied. In addition, VarSome and Priors V2.0 Software were employed for in silico analysis of novel missense variants. A total of 37 *BRCA1* and *BRCA2* pathogenic variants were found in 34 unrelated subjects with a frequency of positive patients of 25.7% (34/132). Twenty-four deleterious variants were detected in *BRCA1* (representing the 64.9% of all identified pathogenic defects) and thirteen (35.1% of all identified pathogenic variants) in *BRCA2* gene. The percentage of patients carrying a variant of unknown significance (VUS) was 7.5% (10/132). In addition, seven novel variants (five in *BRCA2* and two in *BRCA1* gene), never previously reported, were identified. Our approach represents a robust and easy-to-use method for full *BRCA1/2* screening. However, a consistent number of our high-risk families still remained without a satisfying answer. Necessarily, further collective efforts must be directed to a definitive classification of VUSs. The future auspice is that the use of multi-gene panel and more advanced screenings, such as whole exome sequencing and/or RNA seq, in routine diagnostics increases the detection rate.

## 1. Introduction

Germline pathogenic variants (PVs) in the *BRCA1* and *BRCA2* genes confer a significantly increased lifetime risk for the development of both breast and ovarian cancer. For *BRCA1*, the assessed average risk of breast and ovarian cancers ranges from 57 to 65% and 20 to 50%, respectively; for *BRCA2*, the risk ranges from 35 to 57% and 5 to 23%, respectively [1].

The detection of *BRCA1/2* pathogenic variants has important clinical implications for the risk assessment and medical patients’ management. Interventions, such as bilateral mastectomy, salpingo-oophorectomy or annual breast magnetic resonance imaging (MRI) screening, are offered to women carrying *BRCA1/2* PVs in order to reduce cancer risk and to consent early detection. The result of molecular analysis can also influence cancer therapy, principally around the use of platinum agents or poly(ADP-ribose) polymerase (PARP) inhibitors [2]. For these reasons, in the last years, the number *BRCA1/2* genetic tests has increased dramatically. In addition, the development of high-throughput sequencing tools has led many diagnostics centers to use Next Generation Sequencing (NGS)-based platforms for clinical molecular analysis [3,4]. As a consequence, using of these new generation technologies has determined a significant increase in the detection of novel variants. Unfortunately, many of these variants do not provide the evidence of a pathogenic effect (in particular the missense variants or those involved in splicing process) and are classified as variants of uncertain significance (VUSs). The misinterpretation of a VUS has the potential to lead to mismanagement of both the patients and their relatives. For this reason, many efforts should be spent to classify correctly a VUS as deleterious variant rather than as a benign polymorphism [5,6,7]. Currently, to aid genetic counseling of subjects carrying a *BRCA1/2* VUSs, both genetic and functional molecular methods (in-silico and through experimental procedures) are used for the classification of new variants. Genetic analysis is based on the integration of collected family data (co-segregation with disease, co-occurrence with known pathogenic mutations and family history of cancer) into computational models to calculate the likelihood that a VUS is disease-causing [8]. In addition, in silico analysis, predicting the impact of the mutant amino acid on protein folding, functional assays, testing the effect of a VUS on known functions of encoded protein, and gene expression studies are carefully evaluated [9,10,11].

The present study used next-generation sequencing (NGS) technology to determine the prevalence of germline *BRCA1/2* variants in a small cohort of breast/ovarian Italian cancer patients. The obtained results are extensively discussed remarking the novel findings.

## 2. Results

### 2.1. Molecular Analysis of BRCA1/2 Gene

**Figure 1:**
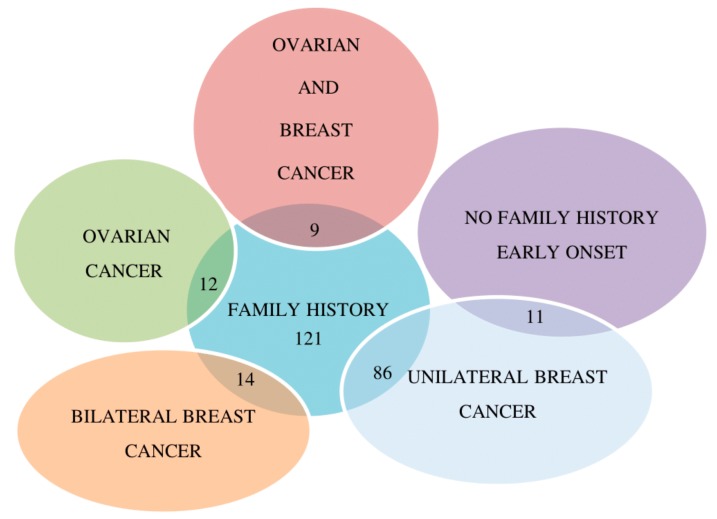
Venn’s diagram regarding the kind of studied patients.

**Figure 2:**
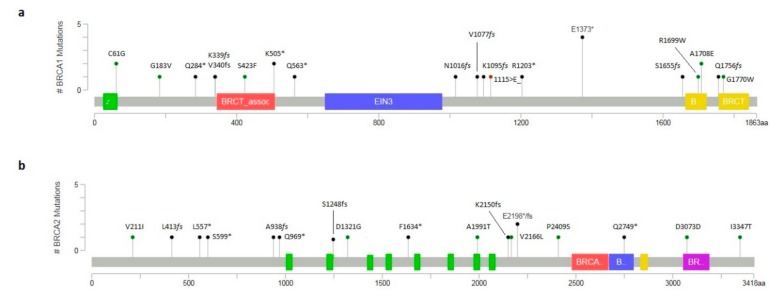
Lollipop plot by MutationMapper reporting all *BRCA1/2* small variants affecting coding region. (**a**) *BRCA1* variants distribution. The c.4117G>T; p.(Glu1373Ter) resulted as the most frequent among detected variants. (**b**) *BRCA2* variants distribution. Two different variants affected the same codon: the p.(Glu2198Ter) and p.(Glu2198AsnfsTer4). Diagram circles are colored with respect to the corresponding variant types. Variant types and corresponding colors are as follows: green for missense and synonymous variants, black for truncating mutations (nonsense and frameshift), brown for in-frame deletions and insertions. *: Stop codon.

We screened *BRCA1* and *BRCA2* genes in 132 Italian patients with early onset or family history of breast and/or ovarian cancers. In particular, 11 patients were women, without any family history of cancer, diagnosed with early onset hormone dependent unilateral breast cancer (median age at diagnosis was 33 years, range 27 to 39). Among the 121 probands with a positive family history of breast and/or ovarian cancers: 86 patients (82 women and four men) were diagnosed with unilateral breast cancer, 14 with bilateral breast cancer (13 women and one man), nine with breast and ovarian cancer and 12 were affected from ovarian cancer (Figure 1). All breast cancers were hormone-receptor positive except 19 triple negative (with negative estrogen receptors, negative progesterone receptors and negative Her2/neu status). All ovarian cancers were serous carcinomas, 17 high-grade and four low-grade. The median age at diagnosis of breast and ovarian cancer was 43 (range 29 to 65) and 52 (range 41 to 66) years, respectively. To better show our findings, Figure 2 reports the distribution and the frequency of all detected variants (with assessed or unknown clinical significant) in *BRCA1* and *BRCA2* genes.

A total of 37 *BRCA1* and *BRCA2* PVs were found in 34 unrelated subjects with a frequency of positive patients of 25.7% (34/132) (Table 1): 24 deleterious variants were present in *BRCA1* (representing the 64.9% of all identified pathogenic defects) and 13 (35.1% of all identified PVs) in the *BRCA2* gene. The percentage of patients carrying a variant of unknown significance (VUS) was 7.5% (10/132). Overall, we identified seven novel variants never previously reported (five in *BRCA2* and two in *BRCA1* gene) (Table 1). 

Among the eleven patients with early onset breast cancer, we identified three women (SA22-18, MV55-17 and LA89-17 in Table 1) carrying known PVs (the c.3607C>T and the c.1016delA mutations in *BRCA1* and the c.1670T>G in *BRCA2*) and two (AM11-17 and TC22-17 in Table 1) with VUS (the c.10040T>C in *BRCA2* and the c.3344_3346delAAG in *BRCA1*).

Fourteen PVs and six VUS were identified among the 67 patients with unilateral hormone-dependent breast cancer and positive family history. In particular, eight PVs (all previously reported including two large rearrangements), two VUS not previously described (the c.5308G>T and the c.1268C>T) and one known VUS, the c.548G>T, were detected in *BRCA1* gene (Table 1). In *BRCA2* gene we identified six deleterious variants (one of these was a novel small insertion, the c.6590_6591insA) and three novel VUS (the c.7225C>T, the c.5971G>A and the c.9219C>T) (Table 1). Two patients (PV86-17 and DPF54-18 in Table 1) carried one *BRCA2* allele with two PVs *in cis*.

Seven PVs and one VUS (all known variants) were detected in eight patients affected from triple negative breast cancer (Table 1). 

Among the patients with bilateral breast cancer, we identified a woman (patient DP97-18 in Table 1) carrying one PV in *BRCA1*(the c.5095C>T) and a small deleterious deletion in *BRCA2* gene (the c.1238delT). In addition, four PVs (one of these corresponding to a novel small deletion, the c.4899_4902delCTTT, causing a premature stop codon at amino acid 1634) and one VUS were identified in *BRCA2* gene while only a deleterious variant was detected in *BRCA1* (Table 1).

Finally, four patients affected from ovarian cancer carried *BRCA1* PVs, while a woman was positive for a deleterious variant in *BRCA2* gene (Table 1). The *BRCA1* c.4964_4982del19, a founder mutation from the southern region of Calabria [12], was detected in a patient (VF31-17 in Table 1) suffering from breast and ovarian cancer and belonging to a family of south Italy origins. 

### 2.2. Novel Variants and In Silico Prediction Analysis

**Figure 3:**
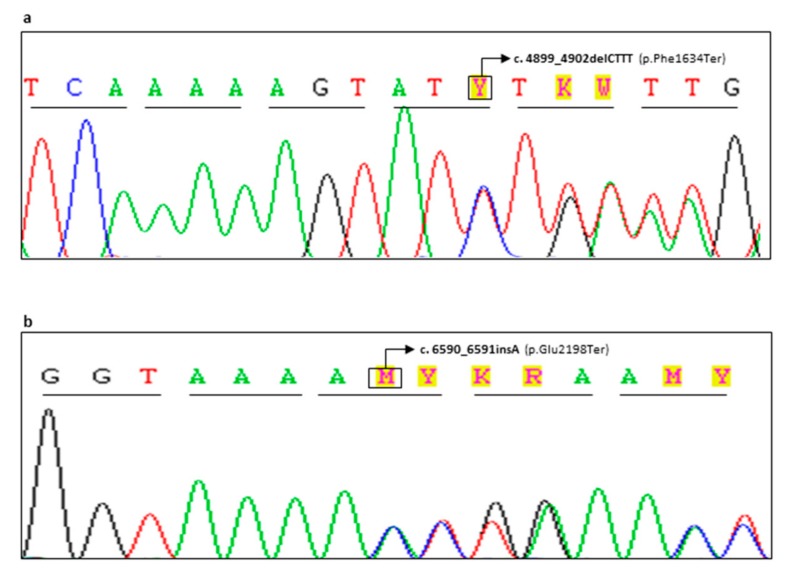
Two novel deleterious variants detected in *BRCA2* gene. (**a**) Electropherogram showing the *BRCA2* c.4899_4902delCTTT variant causing a stop codon at 1634 position within exon 11. (**b**) Electropherogram showing the *BRCA2* c.6590_6591insA variant causing a stop codon at 2198 position within exon 11.

Two novel frameshift variants were identified in *BRCA2* gene. The small frameshift deletion c.4899_4902delCTTT, leading to premature protein termination at codon 1634 (p.Phe1634Ter) within exon 11, was detected in a patient with bilateral breast cancer (patient CG98-18 in Table 1; Figure 3a). The family history was strongly suggestive: the dead father was diagnosed with lung cancer at the age of 66 years, while the paternal aunt died of breast cancer developed at the age of 52 years. Two paternal cousins were suffering from early onset breast cancer and the youngest resulted positive to *BRCA2* genetic screening.

The pathogenicity of the c.4899_4902delCTTT variant is due to the disruption of crucial functional domains (in particular the DNA Binding Domain (DBD), that includes the amino acids 2481-3186) producing a premature truncated non-functional protein. For this reason, according to ENIGMA guidelines [13], it can be classified as Pathogenic (Class 5). 

Similarly, the c.6590_6591insA variant (never reported in queried databases or in literature) showed a clear deleterious effect and it can also be considered as Class 5 variant. This alteration was identified in an early onset breast cancer patient (patient FG65-19 in Table 1; Figure 3b) with three affected sisters, two of these resulting as p.Glu2198Ter carriers, while the third was not tested. 

**Figure 4:**
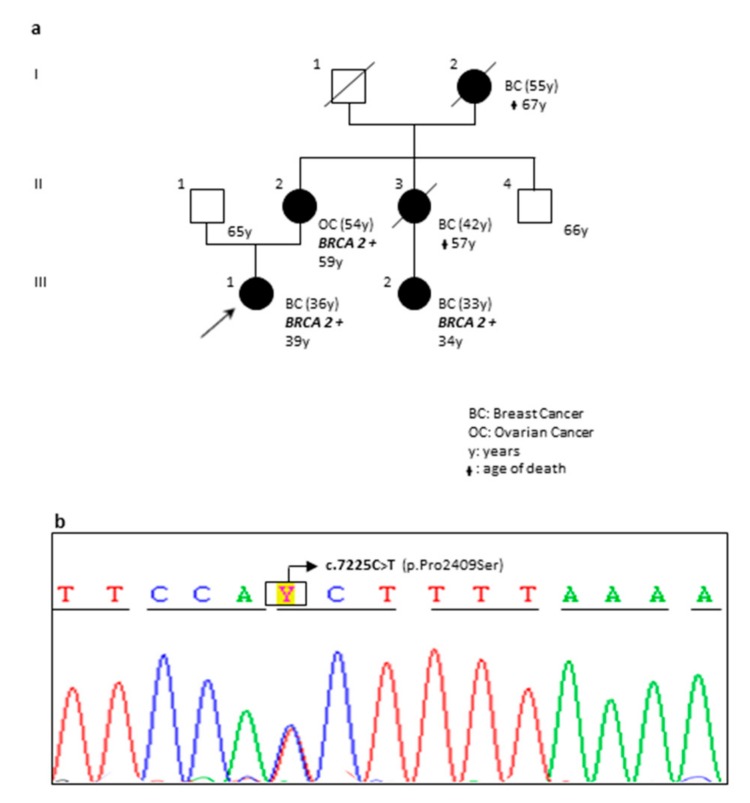
Pedigree and sequencing results of patient carrying the novel *BRCA2* c.7225C>T; p.(Pro2490Ser) variant. (**a**) Pedigree of the proband’s family with three generations depicted. Proband is indicated with an arrow. Black circles indicate affected individuals. Cancer type and age at diagnosis are reported and described as: BC, breast cancer; OV, ovarian cancer. The current age and age of death are also reported. Family members carrying the pathogenic variant are marked with *BRCA2+*. (**b**) Electropherogram showing the *BRCA2* c.7225C>T variant causing the p.(Pro2490Ser) aminoacidic substitution within exon 14.

We identified five novel single nucleotide variants, three in *BRCA2* gene and two in *BRCA1*. The *BRCA2* c.7225C>T (p.Pro2409Ser) variant was detected in a young woman (patient OA78-18 in Table 1) with breast cancer and in two affected relatives (Figure 4a,b). VarSome software classified this substitution as variant of “Uncertain Significance”. However, some evidences supported its potential pathogenicity: in the hot-spot region of the BRCA2 DBD (amino acids 2481-3186), a proved clinical importance was assigned to the most reported missense variants. In addition, the allele c.7225T frequency resulted unknown (no data in GnomAD exomes and GnomAD genomes) and the verdict of computational analysis was “Pathogenic” (pathogenic predictions by DANN, GERP, dbNSFP. FATHMM, LRT, MetaLR, MetaSVM, MutationAssessor and MutationTaster while only PROVEAN in silico analysis resulted as benign). 

**Figure 5:**
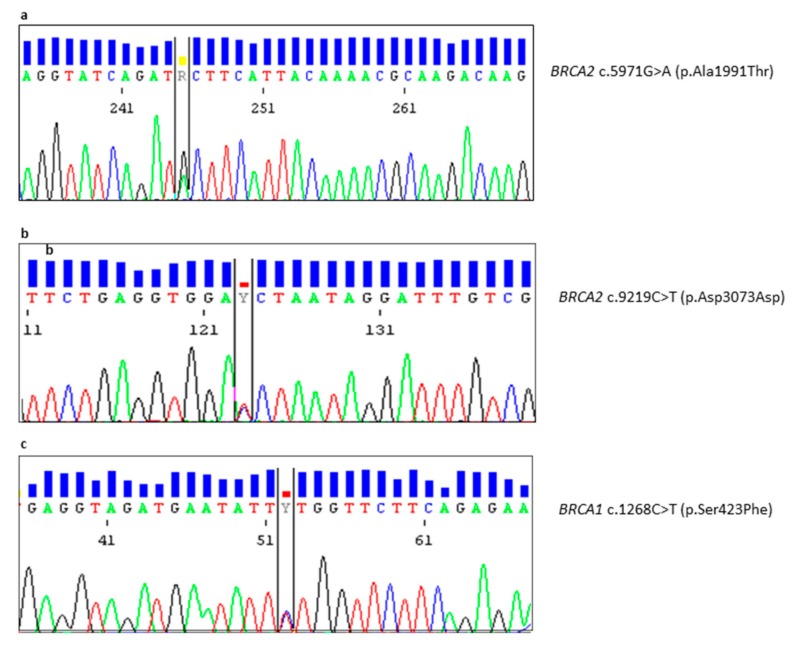
Electropherograms showing novel variants in *BRCA1* and *BRCA2* genes. (**a**) Sequence of the *BRCA2* c.5971G>A variant causing the p.(Ala1991Thr) substitution within exon 11. (**b**) Sequence of the *BRCA2* c.9219C>T; p.(Asp3073Asp) synonymous variant in exon 24. (**c**) Sequence of the *BRCA1* c.1268C>T variant causing the p.(Ser423Phe) substitution in exon 11.

Otherwise, the *BRCA2* c.5971G>A (p.Ala1991Thr) variant, detected in a man with breast cancer at the age of 50 years (patient PM55-16 in Table 1; Figure 5a), was considered by VarSome software as “Likely Benign”. This rare variant (no frequency data on allele c.5971A were available) is located in BRC-7 (1971-2005) motif, where no amino acidic alterations with clinical importance were reported. Noteworthy, the computational verdict was “Benign” because seven benign predictions were provided (coming from dbNSFP, FATHMM, LRT, MetaLR, MetaSVM, MutationAssessor, MutationTaster and PROVEAN) vs one prediction of pathogenicity (from DANN). Finally, the p.Ala1991 position was not reported as conserved (GERP++ rejected substitutions = 0.346 is less than 4). 

The last novel variant detected in *BRCA2* gene was the c.9219C>T (p.Asp3073Asp) (patient MR52-18 in Table 1; Figure 5b) mapping in DBD of BRCA2 protein. This synonymous variant was not predicted as altering the splicing process (in transcript ENST00000544455.1 and NM_000059.3) by Priors analysis (score: 0.02). However, no population frequency data were available for this variant that was considered as “VUS” by VarSome software. 

**Figure 6:**
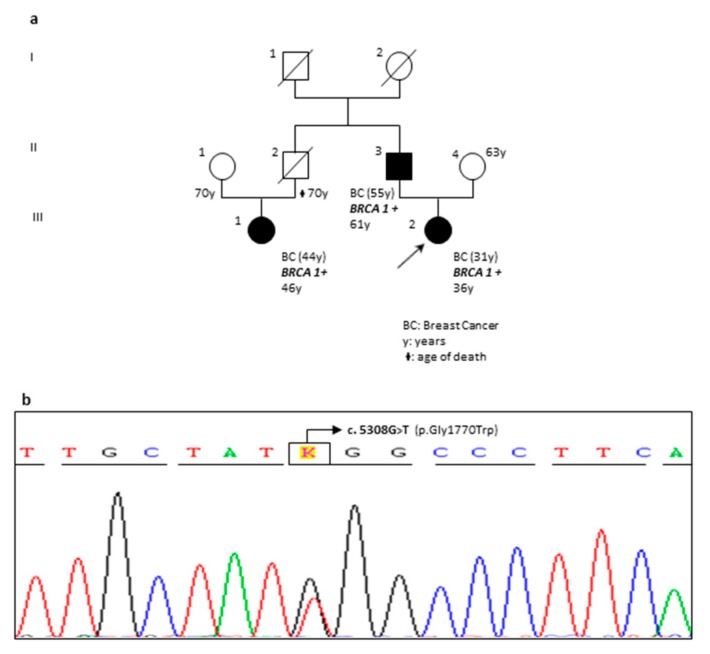
Pedigree and sequencing result of the patient carrying the novel *BRCA1* c.5308G>T; p.(Gly1770Trp) variant. (**a**) Pedigree of the proband’s family with three generations depicted. Proband is indicated with an arrow. Black circles and squares indicate affected individuals. Cancer type and age at diagnosis are reported and described as: BC, breast cancer. The current age and age of death are also reported. Family members carrying the pathogenic variant are marked with *BRCA1+.* (**b**) Electropherogram showing the *BRCA1* c.5308G>T variant causing the p.(Gly1770Trp) aminoacidic substitution within exon 21.

The first novel missense variant found in *BRCA1* gene was the c.5308G>T (p.Gly1770Trp) (patient RR56-18 in Table 1; Figure 6b) located in the hot spot region of the protein (BRTC Domains 1650-1863). Based on the obtained results, VarSome indicated the c.5308G>T as “Likely Pathogenic”. In fact, no frequency data were available on allele c.5308T and the computational analysis verdict was “Pathogenic” because seven pathogenic results (by DANN, GERP, dbNSFP.FATHMM, MetaLR, MetaSVM, MutationAssessor and MutationTaster) *vs* two benign predictions (from LRT and PROVEAN) were obtained. Noteworthy, a further pathogenic evidence was revealed: at the same aminoacidic position, the c.5309G>T (p.Gly1770Val; rs863224765) variant has been previously reported and classified as Likely Pathogenic in both Clin Var and LOVD database. 

Genetic testing was offered to proband’s father, diagnosed with breast cancer at the age of 55, and this screening detected the novel variant in heterozygosis. The same result was obtained analyzing the sequencing data from a paternal cousin with the breast cancer (Figure 6a).

In addition, the *BRCA1* c.1268C>T (p.Ser423Phe) substitution was detected in a woman with breast cancer (patient GH09-19 in Table 1; Figure 5c) and was considered as “VUS” by VarSome software. There were not available frequency data on allele c.1268T and 6 pathogenic predictions (by DANN, dbNSFP.FATHMM, MetaLR, MetaSVM, MutationAssessor and PROVEAN) vs two benign predictions (from LRT and MutationTaster) were reported. Unfortunately, it was not possible to perform genetic testing on the other affected family members.

**Figure 7:**
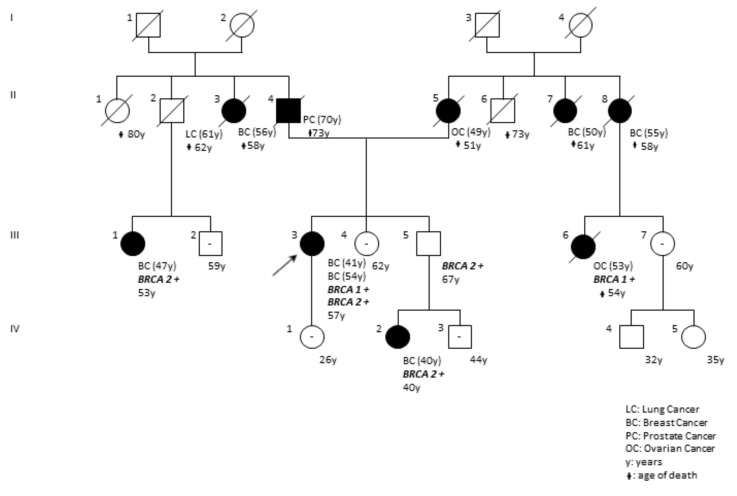
Pedigree of the family with double heterozygosity for *BRCA1* c.5095C>T; p.(Arg1699Trp) and *BRCA2* c.1238delT; p.(Leu413His*fs*Ter17) variants. Proband is indicated with the arrow. Black circles and squares indicate affected individuals. Cancer type and age at diagnosis are reported and described as: BC, breast cancer; OC, ovarian cancer; LC, lung cancer; PC, prostate cancer. The current age and age of death are also reported. Family members carrying a pathogenic variant are marked with *BRCA1+* or *BRCA2+*. Family members negative to genetic testing are indicated with the symbol – within the circles or squares. 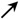: proband.

Finally, Figure 7 describes the pedigree of DP97-18 patient (Table 1) carrying one pathogenic variant in *BRCA1* (the c.5095C>T) and a small deleterious deletion in *BRCA2* gene (the c.1238delT). Genetic testing was also performed in some consenting relatives (III.1, III.5, III.6 and IV.2) and the segregation analysis revealed that the *BRCA2* mutation belonged to paternal familial branch, while the *BRCA1* PV was inherited from the mother. In fact, the subject III.6, a maternal proband’s cousin diagnosed with a metastatic ovarian cancer at the age of 53 and for whom the analysis was performed on formalin-fixed-paraffin-embedded (FFPE) tissue, carried the *BRCA1* c.5095C>T variant in heterozygous status while the III.1 subject, a paternal cousin with a diagnosis of breast cancer at the age of 47, resulted positive for the *BRCA2* small deleterious variant. In this regard, Table 2 reports data regarding all Italian cases related to patients carrying double *BRCA1* and *BRCA2* mutations.

## 3. Discussion

The frequency of *BRCA1/2* PVs in this clinical report was 25.7%, in agreement with previously published studies [4,19,20]. In particular, the c.4117G>T (p.Glu1373Ter) resulted the most frequent PV, representing the 17% (4/24) of all disease-causing alterations identified in *BRCA1* gene. However, this result was not unexpected. In fact, since this variant is often detected in our patients, we are investigating the possible role of this mutation as founder in the Lazio region (data not yet published).

To follow, we discuss the relevant points of our study emphasizing the new findings. As reported in the section results, five novel single nucleotide variants, never previously described in literature or within the main reference databases, have been identified. In order to obtain information about their potential pathogenicity, an accurate evaluation of the patient’s family history was performed (offering genetic testing to all consenting family members) and VarSome software was interrogated. The collected data suggest to very carefully consider two of these variants: the c.5308G>T (p.Gly1770Trp) in *BRCA1* gene and the c.7225C>T (p.Pro2409Ser) in *BRCA2*. The *BRCA1* c.5308G>T (p.Gly1770Trp) was detected in a young woman with breast cancer and in two affected relatives (Figure 6a,b). Three evidences supporting its pathogenicity (results section) emerged from VarSome analysis in addition to the fact that at the same aminoacidic position the p.(Gly1770Trp) variant was previously described with a deleterious significance. Quiles et al. performed the structural analysis of three variants located in the BRCA1 BRCT domain (p.Tyr1703Ser, p.Trp1718Leu and p.Gly1770Val) revealing significant alterations of BRCT structure. The transcription activation (TA) assay showed that these variants dramatically compromise the transcriptional activity of BRCA1 and were classified as likely pathogenic *BRCA1* mutations [21]. At the same time, Bouwman et al. developed a cDNA-based functional assay to classify *BRCA1* unknown variants showing the deleterious effects of the p.Gly1770Val mutation [11]. Overall, these events support the suspect that the p.(Gly1770Trp) may be the disease causing in our family.

The c.7225C>T (p.Pro2409Ser) variant was detected in a young proband diagnosed with breast cancer at the age of 36 and showing a strong family history of cancer. We were able to extend the genetic testing to two affected relatives: the mother with a diagnosis of ovarian cancer and a maternal cousin with early onset breast cancer. Both these women carried the c.7225C>T (p.Pro2409Ser) variant in heterozygous status (Figure 4a,b). In addition, our proband was enrolled in a different study where a multi-gene panel testing for hereditary breast/ovarian cancer predisposition (including the following genes: *ATM*, *BARD1,CDH1,CHEK2*, *PALB2*, *PTEN*, *RAD50*, *TP53*, *PIK3CA*, *RAD51D*, *RAD51C*, *PMS2*, *MLH1*, *MSH2*, *MSH6*, *XRCC2*, *NBN*, *NF1 and STK-11*) was used to assess the mutational status of selected patients. Also in this case, the *BRCA2* c.7225C>T (p.Pro2409Ser) was the only relevant identified alteration. Functional studies are needed in order to investigate the exact role of this novel variant and waiting for further data, we recommend to carefully consider it during the clinical and genetic counselling of carried patients. 

Regarding the others novel identified variants, we believe that: a benign significant could be attributed to the *BRCA2* c.5971G>A (p.Ala1991Thr) and the *BRCA2* c.9219C>T (p.Asp3073Asp) variants, while it is reasonable to consider the *BRCA1* c.1268C>T (p.Ser423Phe) as a VUS. In fact, the collected information on this last variant do not consent to adduce further predictions.

Interestingly, among the patients with bilateral breast cancer, we found a woman (patient DP97-18 in Table 1) carrying one PV in *BRCA1* (the c.5095C>T) and a small deleterious deletion in *BRCA2* gene (the c.1238delT). The presence of double mutations of *BRCA1* and *BRCA2* seems to be an extremely rare event in the general population, although not exceptional in Ashkenazi breast-cancer patients [22]. After a careful examination of the literature, we have identified ten cases of Italian patients with double heterozygosity (Table 2). Most of these studies consists in the description of a single case or of a small number of families [23,24,25,26,27]. A more comprehensive case-control study was published in 2016 using the Consortium of Investigators of Modifiers of *BRCA1/2* (CIMBA) dataset of 32,295 female patients carrying pathogenic *BRCA1/2* variants [27]. From this dataset, 93 trans-heterozygotes (TH) (0.3%) who had inherited deleterious mutations in both *BRCA1* and *BRCA2* genes were identified. The conclusions of this study suggested that *BRCA1* mutation in TH patients may drive the clinical TH phenotype (based on elevated ovarian cancer risk and earlier age of breast cancer diagnosis vs subjects carrying a single pathogenic variant in *BRCA2*) [27]. We think that, although TH subjects would not appear to show a more severe phenotype considering the age of disease onset, cumulative lifetime risk and multiple primary tumors, further studies are needed to individualize the counseling and the clinical management of these patients.

## 4. Material and Methods

### 4.1. Patients

The present study included 132 Italian early onset and familial breast and/or ovarian cancer cases selected by genetic counselling and referred for *BRCA1/2* testing to the Laboratory of Molecular Diagnostics and Genomics of Fondazione Policlinico Universitario A. Gemelli, IRCCS of Rome between 2016 and 2018. 

Single cases of early onset cancer were patients diagnosed with breast cancer before age of 40. For family cases, the likelihood of carrying a *BRCA1/2* pathogenic variants was calculated using BRCAPRO risk assessment tool that is a part of the Cancer Genes software program, version 5.1 (University of Texas Southwestern Medical Center; available at http://www4.utsouthwestern.edu/ breasthealth/cagene) [28]. All patients, after subscription of the consent form, were asked to provide detailed information regarding personal and family history of cancer by an interview. Clinical and pathological characteristics were collected from medical records and pathology reports. The study was conducted in accordance with the Declaration of Helsinki, and the protocol (Protocol ID: 0007205/16) was approved by the Ethics Committee of Università Cattolica del Sacro Cuore, Fondazione Policlinico Universitario Agostino Gemelli (Project ID: ESR14-10185, Approval date: 24 February 2016).

### 4.2. Molecular Analysis

Genomic DNA was obtained using an automatic extractor (MagCore HF16 Plus, Diatech Lab Line, Jesi, Italy); DNA concentration and quality were evaluated by NanoPhotometer™ (Implen, Munchen, Germany) and samples were stored at −20 °C until use. Only DNA meeting following requirements: OD260/280 ratio ≥ 1.7, concentration ≥ 15 ng/μL, no degradation signals visible on agarose gel, were processed.

Library preparation was performed using Devyser BRCA NGS kit (DEVYSER, Hägersten, Sweden), according to the manufacturer’s instructions. The reagent kit V2, 500 Cycles PE, on the Illumina MiSeq System (Illumina, San Diego, CA, USA), and Amplicon Suite Software (SmartSeq, Novara, Italy) were used for sequencing reaction and NGS results interpretation, respectively. The *BRCA1* and *BRCA2* genes reference were: NG_005905.2, NM_007294.3 and NG_012772.3, NM_000059.3. A cut off of 500× coverage was applied to all analyses.

In order to confirm NGS results, PCR-amplified fragments were directly sequenced using BigDye Terminator Cycle Sequencing kit v3.1(Thermo Fisher Scientific) and 3500 Genetic Analyzer (Thermo Fisher Scientific), according to the manufacturer’s instructions. The SeqScape v3.0 software package (Thermo Fisher Scientific) was employed for electropherograms analysis. Variants were named according to Human Genome Variation Society nomenclature (http://www.hgvs.org/mutnomen/). ClinVar (https://www.ncbi.nlm.nih.gov/clinvar/), Leiden Open Variation Database (LOVD; https://www.lovd.nl/) and Evidence-based Network for the Interpretation of Germline Mutant Alleles (ENIGMA; https://enigmaconsortium.org/) Database were used as main reference. Finally, in order to confirm the Copy Number Variation (CNV) prediction results provided by Amplicon Suite Software (SmartSeq, V2, Novara, Italy), the positive samples were investigated using MAQ (Multiplex Amplicon Quantification) assay, as previously reported [29].

### 4.3. In Silico Prediction Analysis

We used VarSome Software (https://varsome.com, V6.7, Lausanne, Switzerland) to investigate the pathogenicity of novel *BRCA1/2* point variants. VarSome is both a powerful annotation tool and search engine for human genomic variants, and a platform enabling the sharing of knowledge on specific variants [30]. If the query is an unknown variant, VarSome provides several evidences supporting the pathogenicity and/or benignity. The verdict is reported according to the American College of Medical Genetics (ACMG) guidelines [31], classifying the variant as: ‘Pathogenic’, ‘Likely Pathogenic’, ‘Likely Benign’, ‘Benign’ or ‘Uncertain Significance’. Population frequency data are taken from gnomAD [14], Kaviar3 [15] and ICGC Somatic [16], while pathogenicity predictions from dbNSFP [17], which compiles prediction scores from 20 different algorithms, and DANN [18]. Finally, Priors V2.0 Software (http://priors.hci.utah.edu/PRIORS/help.php, Salt Lake City, UT, USA) was employed to evaluate the effect of novel synonymous variants on the splicing process.

## 5. Conclusions

In this study, we reported that association of NGS screening with the employment of prediction tools and co-segregation analysis improves the identification of disease causing variants in *BRCA1* and *BRCA2* genes. Our approach identified risk-relevant mutations in 25.7% of analyzed patients. However, we cannot overlook that a consistent number of our high-risk families still remained without a satisfying answer. Unfortunately, the percentage of VUSs in *BRCA1/2* genes is still high and further collective efforts are necessary to reach a definitive classification.

Finally, the auspice is that the use of multi-gene panel in routine diagnostics increases the detection rate, even if the first results from literature data look at more advanced screenings such as whole exome sequencing and/or RNA seq.

## Figures and Tables

**Table 1:** *BRCA1* and *BRCA2* germline variants with assessed or unknown clinical significance detected in Italian patients with breast/ovarian cancer.

Patient	Sex	Gene	Pathogenic Variant (HGVS Variant Sequence ^Δ^)	Pathogenic Variant (HGVS Protein)	Database	Clinical Significance	Proband Cancer/Age of Onset (Relatives)
BF56-18	F	*BRCA1*	c.181T>G (rs28897672)	p.(Cys61Gly)	ENIGMA; Clin Var; LOVD	Pathogenic (Class 5)	Unilateral Breast 30 (Sister *: Breast 42; Mother *: Breast 48)
GU79-18	M	*BRCA2*	c.2808_2811delACAA (rs80359351)	p.(Ala938ProfsTer21)	ENIGMA; Clin Var; LOVD	Pathogenic (Class 5)	Bilateral Breast 65 (Maternal Aunt †: Breast 52, Ovarian Cancer 64; Daughter * asymptomatic 29; Daughter * asymptomatic 32)
CD97-18	F	*BRCA1*	c.5123C>A (rs28897696)	p.(Ala1708Glu)	ENIGMA; Clin Var; LOVD	Pathogenic (Class 5)	Unilateral Breast 51 (Sister twin *: Breast 27; Mother *: Breast 44)
SE53-18	F	*BRCA2*	c.1796_1800delCTTAT (rs276174813)	p.(Ser599Ter)	ENIGMA; Clin Var; LOVD	Pathogenic (Class 5)	Bilateral Breast 43, 49 (Sister *: Breast 37, Father †: Breast 62)
MC52-18	F	*BRCA1*	c.3044dupG (rs80357746)	p.(Asn1016LysfsTer2)	ENIGMA; Clin Var	Pathogenic (Class 5)	TN Breast 35 (Paternal Aunt †: Breast 51, Ovarian Cancer 66; Paternal Cousin *: Breast 36)
RS72-17	F	*BRCA1*	c.4117G>T (rs80357259)	p.(Glu1373Ter)	ENIGMA; Clin Var; LOVD	Pathogenic (Class 5)	TN Breast 40 (Mother *: Bilateral Breast 35, 39; Maternal Grandmother †: Ovarian Cancer 55)
NF20-18	F	*BRCA1*	c.1687C>T (rs80356898)	p.(Gln563Ter)	ENIGMA; Clin Var; LOVD	Pathogenic (Class 5)	TN Breast 36 (Sister *: Ovarian Cancer 44; Brother * asymptomatic 37)
RL94-18	F	*BRCA1*	c.3285delA (rs397509051)	p.(Lys1095AsnfsTer14)	ENIGMA; Clin Var; LOVD	Pathogenic (Class 5)	TN Breast 51 (Maternal Aunt †: Breast 60; Maternal Cousin †: Ovarian Cancer 48)
PV86-17	F	*BRCA2 ▪*	c.2905C>T (rs886038080) c.6447_6448dupTA (rs397507858)	p.(Gln969Ter) p.(Lys2150IlefsTer19)	ENIGMA; Clin Var; LOVD	Pathogenic (Class 5)	Unilateral Breast 49 (Father †: Prostate 57; Paternal Aunt †: Breast 51, Stomach 57; Paternal Uncle †: Lung 55)
PM48-17	F	*BRCA1*	c.850C>T (rs397509330)	p.(Gln284Ter)	ENIGMA; Clin Var; LOVD	Pathogenic (Class 5)	TN Breast 46 (Sister *: Ovarian Cancer 49; Daughter * 21asymptomatic)
AL78-17	F	*BRCA2*	c.8245C>T (rs1135401925)	p.(Gln2749Ter)	ENIGMA; Clin Var	Pathogenic (Class 5)	Unilateral Breast 64 (Daughter *: Ovarian Cancer 36; Son 32 * asymptomatic)
DPF54-18	F	*BRCA2 ▪*	c.631G>A (rs80358871) c.7008-2A>T (rs81002823)	p.(Val211Ile) p.?	ENIGMA; Clin Var; LOVD	Pathogenic (Class 5)	Unilateral Breast 36 (Father *: Prostate 54; Paternal Grandmother†: Ovarian Cancer 55; Brother * 44 asymptomatic)
CS25-18	F	*BRCA2*	c.3744_3747delTGAG (rs80359403)	p.(Ser1248ArgfsTer10)	ENIGMA; Clin Var; LOVD	Pathogenic (Class 5)	Bilateral Breast 44,45, Lung Cancer 53, Pancreas Cancer 66 (Sister *: Bilateral Breast 40,42, Sister *: Bilateral Breast 38,42)
CG98-18	F	***BRCA2***	**c.4899_4902delCTTT**	**p.(Phe1634Ter)**	**Novel Variant**	**Pathogenic (Class 5)**	Bilateral Breast 45,46 (Father†: Lung Cancer 66, Paternal Aunt†: Breast 52, Paternal Cousin*: Breast 36, Paternal Cousin: Breast 40)
DP97-18	F	*BRCA1* *BRCA2*	c.5095C>T (rs55770810) c.1238delT (rs80359271)	p.(Arg1699Trp) p.(Leu413HisfsTer17)	ENIGMA; Clin Var; LOVD Clin Var	Pathogenic (Class 5)	The pedigree of the family is reported in Figure 6.
FG65-19	F	***BRCA2***	**c.6590_6591insA**	**p.(Glu2198Ter)**	**Novel Variant**	**Pathogenic (Class 5)**	Unilateral Breast 29 (Sister *: Breast 37, Sister *: Breast 38, Sister: Breast 40)
MR21-17	F	*BRCA1*	c.1016dupA	(p.Val340GlyfsTer6)	ENIGMA; Clin Var	Pathogenic (Class 5)	Ovarian 46 (Mother †: Breast 44, Maternal Uncle: Lung Cancer 55, Maternal Cousin *: Breast 33)
MC81-17	F	*BRCA1*	c.548G>T (rs1555594081)	p.(Gly183Val)	Clin Var	VUS	Unilateral Breast 48 (Father *: Breast 76)
FM54-17	F	*BRCA1*	c.5468-1G>A (rs80358048)	p.?	Clin Var; LOVD	Pathogenic (Class 5)	TN Breast 44 (Mother: Breast 64, Maternal Aunt: Breast 40; Pancreas Cancer:46)
TM34-17	F	*BRCA1*	c.1513A>T (rs397508877)	p.(Lys505Ter)	ENIGMA; Clin Var; LOVD	Pathogenic (Class 5)	Unilateral Breast 29 (Paternal Grandmother †: Ovarian Cancer 57)
SA22-18	F	*BRCA1*	c.3607C>T (rs62625308)	p.(Arg1203Ter)	ENIGMA; Clin Var; LOVD	Pathogenic (Class 5)	Unilateral Breast 30
GV55-18	F	*BRCA1*	c.5266dupC (rs80357906)	p.(Gln1756ProfsTer74)	ENIGMA; Clin Var	Pathogenic (Class 5)	Ovarian cancer 47 (Mother: Bilateral breast 49,53; Maternal Aunt: Breast 60)
GM55-17	F	*BRCA1*	c.5123C>A (rs28897696)	p.(Ala1708Glu)	ENIGMA; Clin Var; LOVD	Pathogenic (Class 5)	Unilateral Breast 44 (Sister: Breast 46; Father †: Breast 62, Lung Cancer 73)
LA89-17	F	*BRCA2*	c.1670T>G (rs80358452)	p.(Leu557Ter)	Clin Var	Pathogenic (Class 5)	Unilateral Breast 32
LR22-17	F	*BRCA1*	c.(?_-1387-1)_(80+1_81-1)del	p.0?	LOVD	Pathogenic (Class 5)	Unilateral Breast 36 (Mother *: Breast 44; Sister *: Breast 33; Maternal Cousin: Breast 47)
OM41-18	F	*BRCA1*	c.(5193+1_5194-1)_(5277+1_5278-1)del	p.0?	LOVD	Pathogenic (Class 5)	Unilateral Breast 38 (Mother: Breast 56, Colorectal Cancer 48)
QS25-17	F	*BRCA1*	c.181T>G (rs28897672)	p.(Cys61Gly)	ENIGMA; Clin Var; LOVD	Pathogenic (Class 5)	Unilateral Breast 51 (Sister: Breast 44; Paternal Aunt: Breast 40; Paternal Aunt: Breast 46)
SC36-17	F	*BRCA1*	c.1513A>T (rs397508877)	p.(Lys505Ter)	ENIGMA; Clin Var; LOVD	Pathogenic (Class 5)	Bilateral Breast 42,57 (Paternal Aunt: Breast 48; Paternal Cousin: 32)
TG12-17	F	*BRCA1*	c.3228_3229delAG (rs80357635)	p.(Val1077CysfsTer3)	ENIGMA; Clin Var	Pathogenic (Class 5)	TN Breast 45 (Mother: Ovarian Cancer51; Maternal Grandmother †: Breast 58)
KL11-16	F	*BRCA1*	c.4117G>T (rs80357259)	p.(Glu1373Ter)	ENIGMA; Clin Var; LOVD	Pathogenic (Class 5)	Ovarian Cancer 41 (Mother †: Breast 47, Ovarian 62)
VF31-17	F	*BRCA1*	c.4964_4982delctggcctgaccccagaaga (rs80359876)	p.(Ser1655TyrfsTer16)	ENIGMA; Clin Var	Pathogenic (Class 5)	Unilateral Breast 44, Ovarian 48 (Maternal Aunt: Breast 51; Maternal Aunt: Ovarian 55)
PS99-18	F	*BRCA1*	c.4117G>T (rs80357259)	p.(Glu1373Ter)	ENIGMA; Clin Var; LOVD	Pathogenic (Class 5)	Ovarian Cancer 48 (Mother: Ovarian Cancer 52; Sister: Bilateral Breast 36,42)
MV55-17	F	*BRCA1*	c.1016delA (rs80357569)	p.(Lys339ArgfsTer2)	ENIGMA; Clin Var; LOVD	Pathogenic (Class 5)	Unilateral Breast 29
ID38-56	F	*BRCA2*	c.6591_6592delTG (rs80359605)	p.(Glu2198AsnfsTer4)	ENIGMA; Clin Var; LOVD	Pathogenic (Class 5)	Ovarian Cancer 45 (Paternal Grandmother †: Breast 55, Ovarian Cancer 62)
PV22-16	F	*BRCA1*	c.4117G>T (rs80357259)	p.(Glu1373Ter)	ENIGMA; Clin Var; LOVD	Pathogenic (Class 5)	Unilateral Breast 47 (Paternal Cousin: Breast 32; Paternal Cousin: Breast 36)
OA78-18	F	***BRCA2***	**c.7225C>T**	**p.(Pro2409Ser)**	**Novel Variant**	**VUS**	The pedigree of the family is reported in Figure 2.
PM55-16	M	***BRCA2***	**c.5971G>A**	**p.(Ala1991Thr)**	**Novel Variant**	**VUS**	Unilateral Breast 50 (Paternal Aunt: Breast 49; Paternal Aunt: Breast 42)
CA66-17	F	*BRCA2*	c.3962A>G (rs80358645)	p.(Asp1321Gly)	Clin Var	VUS	TN Breast 51 (Mother: Breast 46; Maternal Cousin: Breast 44)
AG55-17	F	*BRCA2*	c.6496G>T (rs750084851)	p.(Val2166Leu)	Clin Var	VUS	Bilateral Breast 45,46 (Maternal Grandmother: Breast 71)
AM11-17	F	*BRCA2*	c.10040T>C (rs587782373)	p.(Ile3347Thr)	Clin Var	VUS	Unilateral Breast 39
TC22-17	F	*BRCA1*	c.3344_3346delAAG	p.(Glu1115del)	Clin Var	VUS	Unilateral Breast 34
MR52-18	F	***BRCA2***	**c.9219c>T**	**p.(Asp3073Asp)**	**Novel Variant**	**VUS**	Unilateral Breast 48 (Mother: Breast 55; Maternal Aunt: Breast 48)
RR56-18	F	***BRCA1***	**c.5308G>T**	**p.(Gly1770Trp)**	**Novel Variant**	**VUS**	The pedigree of the family is reported in Figure 4.
GH09-19	F	***BRCA1***	**c.1268C>T**	**p.(Ser423Phe)**	**Novel Variant**	**VUS**	Unilateral Breast 48 (Sister: Breast 49; Sister: Breast 52)

^Δ^ Variants are numbered in relation to the *BRCA1* and *BRCA2* cDNA reference sequence: NM_007294, NM_000059.3; * Relatives undergone to the genetic testing and resulted carriers of proband’s pathogenic variant. † Dead relative. TN: Triple Negative. ▪ The two variants were *in cis*. The novel variants are reported in bold.

**Table 2:** Italian patients carrying pathogenic variants at both *BRCA1* and *BRCA2*. Data from literature review.

*BRCA1* Pathogenic Variant (HGVS Sequence ^Δ^/Protein)	*BRCA2* Pathogenic Variant (HGVS Sequence ^Δ^/Protein)	Sex	Inheritance		Proband Cancer (Age at Diagnosis)	Reference
			Mother	Father		
c.4285_4286insG; p.(Tyr1429Ter)	c.7738C>T; p.(Gln2580Ter)	F	ND	ND	Breast37	[14]
c.5266dupC; p.(Gln1756ProfsTer74)	c.5796_5797delTA; p.(His1932GlnfsTer12)	F	*BRCA1*	*BRCA2*	Breast38, Ovarian42	[15]
c.835delC; p.(His279MetfsTer19)	c.8195T>G; p.(Leu2732Ter)	F	ND	ND	Breast43	[16]
c.3916_3917delTT; p.(Leu1306AspfsTer23)	c.5379delT; p.(Asn1793LysfsTer2)	F	WT	ND	Breast30, Ovarian36	[15]
c.1687C>T; p.(Gln563Ter)	c.6469C>T; p.(Gln2157Ter)	F	ND; ND	ND	Breast46, Ovarian58	[16]
c.2405_2406delTG; p.(Val802GlufsTer7)	c.4284dupT; p.(Gln1429SerfsTer9)	F	ND	ND	Breast and Ovarian52	[16]
c.547+2T>A	c.2830A>T; p.(Lys944Ter)	F	WT	*BRCA1/BRCA2*	Breast35	[17]
c.213-12A>G	c.7180A>T; p.(Arg2394Ter)	F	-	-	Breast-	[18]
c.3228_3229delAG; p.(Gly1077AlafsTer8)	c.9253dupA; p.(Thr3085AsnfsTer26)	F	-	-	Breast-	[18]
c.3477_3480delAAAG; p.(Ile1159MetfsTer50)	c.9401delG; p.(Gly3134AlafsTer29)	F	-	-	Ovarian-	[18]
c.5095C>T; p.(Arg1699Trp)	c.1238delT; p.(Leu413HisfsTer17)	F	*BRCA1; BRCA2*	*BRCA2*	Bilateral Breast41,54	This report

^Δ^ Variants are numbered in relation to the *BRCA1* and *BRCA2* cDNA reference sequence: NM_007294, NM_000059.3. F: Female, ND: Not Determined, WT: wild type, -: not provide information.
